# Personalized training and orthopedic inserts bring significant benefits in the posturographic assessment of patients with early gonarthrosis – a randomized controlled trial

**DOI:** 10.1038/s41598-026-56771-4

**Published:** 2026-06-12

**Authors:** Amanda Maria Kostro, Janusz Dzięcioł, Mariusz Baumgart, Bartosz Bagiński, Agnieszka Sozańska, Zofia Dzięcioł-Anikiej

**Affiliations:** 1 Department of Personalized Physiotherapy of the Musculoskeletal System, Faculty of Health Sciences, Medical University of Bialystok, Jana Kilińskiego 1, 15-089 Bialystok, Poland; 2 Department of Human Anatomy, Faculty of Medicine, Medical University of Bialystok, Jana Kilińskiego 1, 15-089 Bialystok, Poland; 3 Department of Normal Anatomy, Faculty of Medicine, Collegium Medicum in Bydgoszcz, Nicolaus Copernicus University in Torun, Łukasiewicza 1, 85-821 Bydgoszcz, Poland; 4 Faculty of Health Sciences and Psychology, Collegium Medicum, University of Rzeszow, Rejtana 16C, 35-959 Rzeszow, Poland; 5 Health, Functioning and Disability Research Laboratory, University Research and Development Center in Health Sciences, Warzywna 1a, 35-310 Rzeszów, Poland; 6 Physical Activity Research Laboratory of the Clinical Research Center, Medical University of Bialystok, Jana Kilińskiego 1, 15-089 Bialystok, Poland

**Keywords:** Posturography, Gonarthrosis, Rehabilitation, Stabilization, Foot inserts, Medical research, Plant sciences

## Abstract

Knee osteoarthritis (KOA) results from cartilage degeneration and abnormal load distribution, leading to impaired biomechanics and postural control. Foot abnormalities such as hypermobility, flat feet, and pronation—common in KOA—may contribute to the degeneration process of the knee joint structures. Stabilization and proprioceptive exercises, as well as orthopedic inserts, can modify these factors by improving muscle function and redistributing load. However, studies directly comparing their effectiveness using objective assessment methods are lacking. Postural control plays a key role in KOA because it reflects the integration of balance systems. The aim of the study was to evaluate the effect of these interventions and their combination on postural balance in centre of pressure (COP) and clinical outcomes (KOOS – knee injury and osteoarthritis outcome score) in patients with early KOA. Participants were randomly divided into four groups: “Exercises” (n = 43), “Orthopaedic insoles” (n = 43), “Mixed” (n = 39), “Control” (n = 41). During the qualification process, a posturographic examination was performed on a force platform with eyes open and closed in a standing position. Participants were also assessed using the KOOS questionnaire. The obtained data were analyzed in Statistica 13 using the Wilcoxon signed-rank test and the Kruskal–Wallis ANOVA test. A significance level of p = 0.05 was used. Posturographic parameters such as sways length and mean speed with eyes open and closed improved, and the ellipse area of the center of gravity decreased in the mixed group (p = 0.001). The largest clinical effects were observed for sways length with eyes open (d = 2.512) between the control and exercise groups and for sways length with eyes closed (d = 2.172), ellipse area with eyes open (d = 1.361), and mean speed with eyes closed (d = 2.193) between the control and mixed groups. Quality of life (QoL) and functioning improved statistically, and significant between-group differences were found for pain, symptoms, physical activities of daily living (ADL), sports/recreation, and QoL (p < 0.001). However, there were no significant differences between the exercise group and the orthopedic insole group in terms of symptoms, ADL, sports/recreation, and QoL. A significant clinical effect was observed on the KOOS pain score between the insoles and exercise groups (d = 1.122). The combined use of orthopedic insoles and exercises is the most effective method of rehabilitation.

Trial registration: The study was registered in the international Research Registry database (https://www.researchregistry.com/) under registration number researchregistry11661.

## Introduction

Knee osteoarthritis (KOA, gonarthrosis) is one of the most common musculoskeletal disorders, affecting primarily middle-aged and older adults. Its development is associated with the degeneration of joint cartilage, which—despite its high resistance to mechanical stress—is damaged by abnormal force distribution and chronic overload, leading to metabolic disorders^1^. Additionally, factors such as obesity, inflammation, and lower limb axis abnormalities contribute to disease progression. KOA is a significant public health problem, being one of the leading causes of chronic pain and disability. Clinical symptoms include pain, reduced muscle strength, limited range of motion (ROM), impaired proprioception, and impaired performance in activities of daily living (ADLs)^2^. Uneven cartilage reduction coexists with pathological alteration of the lower limb axis, leading to impaired muscle function, limited range of motion, and pain. In turn, pain in the affected joint transfers load to the healthy limb, which disrupts posture and predisposes to balance deficits^3,4^. Consequently, both static and dynamic stability are impaired, and the risk of falls is increased^5^. Therefore, proprioceptive deficits are observed even in the early stages of KOA, as confirmed by posturography^6^. Muscles are sensitive to such changes and therefore unable to properly stabilize the joint^7,8^, and the load they generate is often directed towards the medial and anterior aspects of the knee joint^9^. Pain-related limitation of physical activity promotes further joint degeneration^7^.

Furthermore, osteoarthritis coexists with an elevated body mass index (BMI) and correlates with an increased systemic inflammatory response. Studies show that inflammatory processes are associated with reduced muscle strength and can also occur in healthy adults^10^. In turn, rapid weight loss can lead to joint destabilization and loss of muscle and bone mass, highlighting the importance of implementing sustainable weight management strategies^11^.

Lower limb biomechanical abnormalities, including foot malalignment (e.g., pronation, flat feet), common in KOA, may contribute to the degenerative process of knee joint structures^12–14^.

Due to the multifactorial etiology of KOA, conservative treatment focuses on modifiable risk factors. Therefore, exercise programs aimed at reducing symptoms and improving or maintaining physical function and postural control play a key role in KOA treatment^15–17^. Proprioceptive and stabilization exercises are particularly important, although the quality of evidence remains moderate^18–20^. Resistance exercises are also useful, particularly in pain management^21^, but they should incorporate elements that stabilize and relieve joints by strengthening muscles and ligamentous structures, aiming to induce hypertrophy of periarticular muscles, which allows for the effective distribution of forces acting on joint surfaces^22^. Core stability training can further improve postural control and motor coordination^23^. At the same time, orthopedic insoles constitute an important conservative treatment method, influencing foot alignment correction and load redistribution, which can lead to improved postural stability^24–28^.

Despite extensive evidence of the effectiveness of exercises^18–21,23^ and insoles^24–28^ in patients with early osteoarthritis, a significant research gap exists. There is a lack of randomized, controlled trials directly comparing these interventions in patients with early KOA, particularly those using posturography. Previous studies have often relied on comparisons with healthy controls, limiting the ability to assess the true effectiveness of interventions in clinical populations. Although research methodology often utilizes posturography to assess balance impairments, for example, in patients with KOA^6,29–31^ or after knee surgery^32,33^, and only a few reports have included this method in the evaluation of insole use^34^ or rehabilitation^26^, this highlights the limited use of objective balance assessment in comparative interventional studies.

Postural control is a key component of the functioning of patients with KOA, reflecting the integration of sensory and motor systems responsible for maintaining balance. Posturography enables an objective and sensitive assessment of these processes by analyzing center of pressure (COP) parameters such as trajectory length, velocity, and sway area.

This study compared the effectiveness of individualized medical training, including stabilization and proprioceptive exercises, and the use of individually fitted orthopedic insoles, compared to a control group of patients with KOA. The main aim of the study was to evaluate the effect of combining stabilization exercises with individually fitted orthotic inserts on postural balance in patients with early medial KOA, as assessed by center of pressure (COP) parameters on a force platform.

The secondary objective was to evaluate the impact of the intervention on patient-reported clinical outcomes, including pain, symptoms, functioning in daily life, sports and recreational activity, and quality of life, as measured by the KOOS scale (knee injury and osteoarthritis outcome score).

The null hypothesis assumed no significant differences in changes in postural balance parameters and KOOS scores between the study groups over time. The alternative hypothesis assumed significant differences between the groups, with particular emphasis on greater improvement in the group receiving the combined intervention (exercises + orthopedic insoles).

## Methods

### Trial design

The study was a prospective, randomized, controlled trial (RCT) with four parallel arms and was conducted according to CONSORT 2025 guidelines. The aim of the study was to evaluate the effectiveness of combining stabilization exercises with custom-fit orthotics in patients with early medial KOA.

### Ethics and dissemination

The study protocol was approved by the Bioethics Committee of the Medical University of Białystok (no. APK.002.86.2024) and conducted in accordance with the principles of the Declaration of Helsinki. All participants provided written, informed consent to participate in the study after receiving full information about its course, aims, and potential risks. Participants’ personal data were processed anonymously, in accordance with personal data protection regulations (GDPR). Patients were informed about the personal data that would be used and reported in the study, including age, BMI, and gender. Each participant also provided their name and surname at the first meeting (including by legibly signing a consent form). These data were ultimately not used in the subsequent stages. Patients were coded sequentially according to the following formula: OA1, OA2…

### Trial registration and data availability

The study was registered retrospectively in the international Research Registry database (https://www.researchregistry.com/) under the registration number researchregistry11661. Date of first publication: November 18, 2025. Registration was conducted in accordance with the principles of transparency of clinical trials and the CONSORT 2025 and SPIRIT recommendations.

### Trial setting

The study was conducted in the orthopedic and rehabilitation clinic and in the biomechanics of movement laboratory from February 2024 to February 2025. All measurements and interventions took place in a controlled outpatient setting.

### Recruitment

Participants were recruited in the orthopedic and rehabilitation clinic from February 2024 to February 2025. Patients were informed about the study by orthopedic doctors and physiotherapists and then referred for qualification to participate in the project.

### Eligibility criteria

The study included patients diagnosed with gonarthrosis (KOA) according to the American College of Rheumatology (ACR) criteria, with radiographic Kellgren–Lawrence stage 1–2, with medial compartment involvement confirmed by clinical and imaging examination, aged 40–60 years, in stable clinical condition, free of acute pain, and able to ambulate independently.

Written, informed consent was required for participation in the study and acceptance of custom orthopedic inserts if assigned to the appropriate group. The study excluded individuals with advanced radiological changes (Kellgren–Lawrence ≥ 3), significant ligament instability or deformity of the lower limb axis, knee surgery in the last six months, neurological or vestibular diseases affecting balance, other orthopedic conditions affecting stability, severe obesity (BMI > 35 kg/m^2^), participating in other rehabilitation programs in the period of last three months, or not consenting to participate.

### Intervention and comparator

Participants were randomly assigned to one of four groups: (1) stabilization exercises + custom orthotics, (2) stabilization exercises, (3) custom orthotics, or (4) a control group without intervention.

#### Group I (mixed: exercise + orthopeadic insoles)

Participants completed a six-week stabilization exercise program and wore individually fitted orthopedic insoles. The exercises included balance, proprioceptive, and strengthening training the main muscle groups—the trunk and lower limbs (training core stabilization of body). Sessions were held three times a week, 45 min each, under the supervision of a physiotherapist, using the same protocol. The insoles were individually tailored based on a podoscopic examination and gait analysis. They included supination elements supporting the arch of the foot, correcting for the medial hindfoot (heel valgus), and a forefoot pronation element. Patients wore them daily for a minimum of 6 h, starting with 1 h on day 1 and gradually increasing the wearing time by 1 h each subsequent day.

#### Group II (exercise)

Participants completed the same exercise program without the use of orthopedic insoles.

#### Group III (orthopeadic insoles)

Participants used only custom orthopedic insoles for six weeks, without participating in an exercise program.

#### Group IV (control)

Participants received no intervention during the study and maintained their current level of physical activity. All groups were assessed before and after the intervention. It does not apply to combined care or additional options beyond the treatment being studied.

The exercise program lasted six weeks and included three sessions per week led by a physiotherapist, focusing on proprioceptive, balance, and strengthening exercises main muscle groups of the trunk and lower limbs. The orthotics were individually administered by a podiatrist based on foot and gait analysis. Participants in the control groups received no intervention during this period.

### Randomization

Randomization was conducted using a computerized random number generator using a simple 1:1:1:1 randomization scheme. The allocation sequence was prepared by a researcher independent of the recruitment and outcome assessment processes. Information about allocation was placed in numbered, sealed, and opaque envelopes, which were opened only after the participant’s baseline assessment was completed. This ensured allocation concealment and limited the risk of selection bias.

### Blinding

The study was conducted using a single-blind design, in which the individuals performing the measurements and data analysis had no information about the participants group assignment. Participants were not fully blinded due to the nature of the intervention (exercise and orthotic inserts), but they were not informed of the study hypothesis or the expected effects of each intervention. The balance and questionnaire scorers were independent and did not participate in the implementation of the exercise program.

### Participant timeline

The intervention lasted six weeks. All endpoints were reassessed after the intervention. The total duration of participation per a participant in the study was approximately six weeks, including screening visits and final measurements.

### Measurement tools and assessment methods

Objective and subjective measurement methods were used to evaluate the effects of the intervention.

1. Postural balance was assessed using the FreeMED BASE posturographic platform (Sensor Medica FreeSTEP—V.1.6.004, Italy), which records the displacement of the center of pressure (COP) in the X–Y coordinate system. Recording was performed at a frequency of 100 Hz for 30 s in a standing position, with eyes open and feet hip-width apart. Three main parameters were analyzed: the COP path length, the average COP velocity, and the area of the ellipse defining 95% of the COP excursions (COP ellipse area). Each measurement was repeated three times, and the average of three trials was used for analysis. Measurements were taken at the same time of day and under the same environmental conditions to ensure reproducibility. Studies have demonstrated high reliability of measurements obtained using stabilographic platforms, with repeatability coefficients (ICC) ranging from 0.70 to 0.95, depending on the measurement conditions and the parameter being analyzed. The COP trajectory length is characterized by particularly good repeatability in test–retest measurements. The validity of these measures was confirmed by their ability to differentiate between healthy individuals and patients with balance disorders and to detect changes following therapeutic interventions. Stabilographic platforms are considered the gold standard for quantitative assessment of postural control in biomechanical and clinical studies. The measurement procedures in this study followed standardization recommendations for stabilographic studies, which increases the reliability and comparability of the obtained results^35,36^.
2. Functional assessment and quality of life were performed using the KOOS (Knee Injury and Osteoarthritis Outcome Score) questionnaire, in its validated Polish language version^37,38^. The tool comprises five subscales: pain, symptoms, function in daily activities, sports and recreational activity, and quality of life. Scores on each subscale were converted to a scale from 0 to 100, with higher scores indicating better function and reduced symptom severity. The questionnaires were completed independently by participants under the supervision of an evaluator, before and after the intervention.

### Outcomes

The primary outcome was the change in postural balance parameters, assessed by center of pressure (COP) trajectory length, COP velocity, and COP ellipse area measured on a posturographic platform. The secondary endpoint was change in KOOS subscale scores (pain, symptoms, function, sports/recreation, quality of life). Assessments were performed before and after the intervention.

### Harms

Adverse events, such as increased pain, discomfort during exercise, or skin irritation at the site of contact with the inserts, were monitored throughout the study. No serious adverse events requiring discontinuation of the intervention were reported.

### Sample size

The sample size was determined based on a power analysis aimed at detecting a significant difference between groups in COP trajectory length of at least 10% (a minimum difference of 10% between groups was assumed because this value exceeds the typical measurement variability of COP parameters reported in reliability studies and falls within the range of changes considered clinically important in the assessment of postural control^35,36^), with a significance level of α = 0.05 and power (1–β) = 0.8. The minimum number of participants in each group was 40, including a 10% allowance for potential dropouts.

### Statistical methods

Statistical analysis was performed using Statistica 13.0 software (StatSoft, Poland). All measurements were taken twice—before and after the intervention—at the same time of day and using the same measurement equipment to minimize the influence of external factors on the results.

The Shapiro–Wilk test assessed the normality of the data distribution. Nonparametric methods were used when the assumptions of parametric tests were not met.

For intragroup comparisons, i.e., assessing changes in the same group of patients before and after the treatment, the Wilcoxon signed-rank test was used. This nonparametric method compares two dependent samples and is an alternative to the Student’s t-test for paired samples when the distribution of differences between observations is not normal. This test is particularly useful when analyzing the results of measurements taken before and after a given intervention in the same participants.

The Kruskal–Wallis ANOVA test, a nonparametric equivalent of one-way analysis of variance, was used to assess differences between treatment groups. This test allows for the comparison of medians across more than two independent groups when the assumptions of normality or homogeneity of variance are not met. If significant differences were found, post-hoc comparisons were performed using Dunn’s test to determine which groups had statistically significant differences. Additionally, the effect size was calculated using Cohen’s d, which expresses the standardized difference between two means:

$$d = \frac{{\overline{X}_{1} - \overline{X}_{2} }}{{SD_{{{\mathrm{pooled}}}} }}$$

Effect sizes were interpreted according to Cohen’s classification: small effect size (d = 0.2), medium effect size (d = 0.5), and large effect size (d ≥ 0.8). All statistical tests were two-sided, and the level of statistical significance was set at p < 0.05. Results were presented as means ± standard deviation (SD) or medians with interquartile range (IQR), depending on the nature of the data and whether the assumptions of normal distribution were met.

Multivariate analysis using the general linear model (GLM) was performed to assess the potential influence of age and BMI as covariates.

The study had no missing data or loss to follow-up. All randomized participants were included in the analysis according to the intention-to-treat (ITT) principle. Participants who did not meet the inclusion criteria were excluded at recruitment, prior to randomization, and were not included in the statistical analyses.

The analysis methods were specified a priori in the study protocol, while post hoc analyses were exploratory in nature. Adjustment for multiple testing was made when performing post hoc tests comparing groups by applying Bonferroni correction for each variable.

## Results

**Fig. 1:**
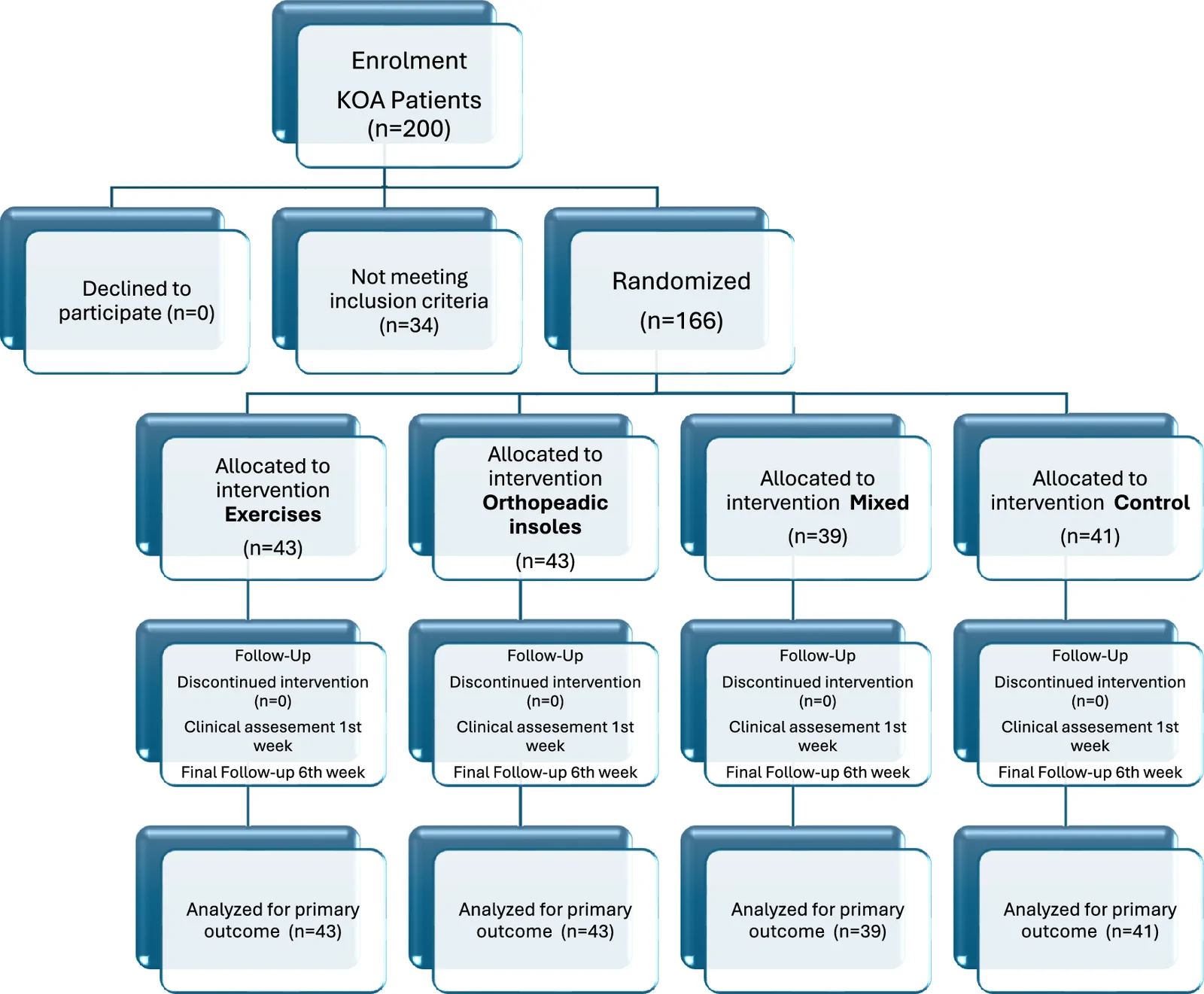
CONSORT 2025 Flow Diagram of the process through the phases of a randomized trial of three groups.

A total of 200 patients with KOA were assessed for eligibility. No participants declined to participate, and 34 did not meet the inclusion criteria. A total of 166 participants were randomized into four groups: Exercises (n = 43), Orthopaedic insoles (n = 43), Mixed intervention (n = 39), and Control (n = 41). No participants discontinued the intervention or were lost to follow-up. All participants completed clinical assessments at week 1 and week 6. All randomized participants were included in the primary outcome analysis (Exercises n = 43; Orthopaedic insoles n = 43; Mixed n = 39; Control n = 41) (Fig. 1).

The demographic structure of the groups is presented in Table 1.

**Table 1:** Demographic structure of groups.

		Variable	n	M	CI (95%)		Me	Min	Max	Q1	Q3	SD
Control	Women	Age	25	47,56	44,65	50,47	45,00	40,00	60,00	41,00	55,00	7,06
		BMI	25	26,98	24,58	29,38	25,69	19,38	38,67	23,42	33,06	5,81
	Men	Age	16	45,13	42,50	47,75	44,50	40,00	58,00	41,50	48,00	4,92
		BMI	16	27,28	24,84	29,73	26,87	18,94	34,87	24,63	30,10	4,59
Orthopeadic insoles	Women	Age	35	52,74	50,65	54,84	56,00	40,00	60,00	47,00	57,00	6,10
		BMI	35	28,54	26,90	30,19	27,41	18,52	39,79	26,12	30,48	4,79
	Men	Age	8	51,13	45,26	56,99	54,50	41,00	59,00	44,00	56,00	7,02
		BMI	8	30,58	28,55	32,61	30,25	28,09	35,35	28,56	31,80	2,43
Exercises	Women	Age	33	54,24	52,93	55,55	54,00	42,00	60,00	53,00	56,00	3,69
		BMI	33	25,41	24,22	26,60	24,22	20,07	34,89	23,44	27,64	3,37
	Men	Age	10	46,80	41,49	52,11	43,00	41,00	60,00	42,00	55,00	7,42
		BMI	10	29,48	25,60	33,36	28,08	23,41	37,40	24,69	36,39	5,42
Mixed	Women	Age	26	54,69	52,14	57,24	56,00	40,00	60,00	54,00	60,00	6,31
		BMI	26	26,85	25,21	28,50	25,32	21,16	36,44	23,80	28,34	4,07
	Men	Age	13	51,15	46,34	55,97	54,00	40,00	60,00	44,00	59,00	7,97
		BMI	13	29,36	26,13	32,58	28,53	22,72	37,51	24,84	32,98	5,33

*BMI* body mass index, *n* numbers, *M* mean, CI (95%) 95% confidence interval, *Me* median, *Min* minimum, *Max* maximum, *Q1* quartile 1, *Q3* quartile 3, *SD* standard deviation.

Analysis of the results showed that statistically significant changes in posturographic parameters occurred only in the groups that received any type of therapeutic intervention (Table 2). In both the stabilization exercise groups, the group using individual orthopedic insoles, and the combined group (exercise + insoles), a decrease in the center of pressure (COP) path length and the mean velocity of COP sway with eyes open and closed was observed (p < 0.05). These changes indicate improved postural stability after the completion of therapy.

**Table 2:** Assessment of individual postsurographic parameters before and after therapy in individual study groups.

	Group	Before										After 6 weeks										p*
		n	M	CI (95%)		Me	Min	Max	Q1	Q3	SD	n	M	CI (95%)		Me	Min	Max	Q1	Q3	SD	
Sway length OE (mm)	Control	41	1078,89	1009,78	1147,99	1034,27	758,35	1733,30	913,30	1201,25	218,95	41	1439,65	1307,69	1571,60	1370,03	907,10	2607,08	1232,96	1523,89	418,06	(*) p < 0,001
	Exercises	43	2530,98	2327,33	2734,63	2486,19	1495,49	4867,82	2063,81	2763,47	661,73	43	1515,04	1361,99	1668,10	1490,05	862,79	3238,01	1102,68	1841,81	497,34	(*) p < 0,001
	Orthopeadic insoles	43	2648,26	2456,92	2839,60	2503,27	1788,50	4276,85	2192,20	3187,33	621,73	43	1402,08	1249,87	1554,28	1366,64	211,78	2607,08	1120,15	1666,76	494,56	(*) p < 0,001
	Mixed	39	3003,13	2690,08	3316,18	2694,33	1462,01	4960,47	2258,54	4012,55	965,73	39	1353,97	1211,27	1496,68	1349,59	862,83	2337,32	943,29	1809,31	440,23	(*) p < 0,001
Sway length CE (mm)	Control	41	1153,66	1068,54	1238,79	1118,76	763,45	1692,02	950,00	1285,25	269,69	41	1559,38	1351,82	1766,93	1410,53	859,80	3641,43	1214,95	1723,27	657,57	(*) p < 0,001
	Exercises	43	2421,79	2221,04	2622,54	2341,23	1334,03	4770,09	2046,50	2754,04	652,30	43	1595,04	1441,68	1748,39	1596,06	875,53	3376,67	1206,42	1724,16	498,31	(*) p < 0,001
	Orthopeadic insoles	43	2547,54	2400,48	2694,61	2530,15	1612,37	3590,54	2341,23	2755,46	477,87	43	1565,81	1389,95	1741,66	1399,29	763,45	3641,43	1199,96	1881,76	571,41	(*) p < 0,001
	Mixed	39	2870,60	2577,16	3164,04	2674,61	1605,80	5693,17	2269,01	3242,64	905,22	39	1416,08	1260,95	1571,21	1285,25	802,69	2393,80	986,07	1956,87	478,56	(*) p < 0,001
Ellipse area OE (mm^2^)	Control	41	149,46	83,77	215,16	80,24	14,20	909,75	55,23	111,40	208,13	41	295,72	191,84	399,59	164,59	31,04	1318,91	133,20	247,69	329,10	(*) p < 0,001
	Exercises	43	156,35	121,09	191,61	108,51	23,56	423,05	70,50	192,89	114,57	43	180,44	106,98	253,91	95,45	24,79	1318,91	67,24	183,28	238,72	0,904
	Orthopeadic insoles	43	189,41	117,28	261,53	103,77	23,91	1098,68	62,49	182,12	234,37	43	150,12	94,18	206,07	104,99	27,62	909,75	62,49	144,89	181,77	0,205
	Mixed	39	179,43	114,88	243,97	96,89	23,56	739,19	68,55	156,70	199,11	39	92,74	47,58	137,91	76,34	14,20	909,75	43,10	97,77	139,33	(*) p < 0,001
Ellipse area CE (mm^2^)	Control	41	118,17	89,21	147,14	90,73	11,59	414,07	58,47	163,80	91,77	41	168,08	130,14	206,03	155,54	9,83	568,94	93,93	209,17	120,22	0,045
	Exercises	43	108,96	79,81	138,12	79,73	3,55	417,02	49,51	139,24	94,73	43	112,25	87,47	137,04	87,84	31,26	352,39	49,51	165,50	80,54	0,433
	Orthopeadic insoles	43	157,32	108,44	206,19	89,29	3,55	648,27	45,87	214,35	158,80	43	117,93	86,24	149,62	97,17	29,76	499,70	62,89	128,93	102,97	0,398
	Mixed	39	152,57	109,11	196,04	90,60	12,66	461,16	56,93	214,35	134,09	39	113,27	82,91	143,63	89,29	31,26	499,70	55,23	145,99	93,65	0,061
Mean speed OE (mm/s)	Control	41	21,23	19,87	22,59	20,31	14,99	34,14	17,90	23,61	4,32	41	28,40	25,80	30,99	27,02	17,75	51,18	24,34	29,99	8,22	(*) p < 0,001
	Exercises	43	49,90	45,90	53,90	49,21	29,31	95,92	41,27	54,24	13,00	43	29,86	26,82	32,90	29,15	16,99	64,13	21,68	36,69	9,88	(*) p < 0,001
	Orthopeadic insoles	43	52,19	48,48	55,90	49,27	35,47	83,60	43,19	62,61	12,06	43	28,58	25,69	31,48	26,86	14,99	51,18	22,05	34,59	9,41	(*) p < 0,001
	Mixed	39	59,14	53,00	65,29	53,27	28,73	97,48	44,23	78,80	18,96	39	26,71	23,89	29,53	26,60	16,85	46,16	18,53	35,40	8,70	(*) p < 0,001
Mean speed CE (mm/s)	Control	41	22,57	20,89	24,25	21,75	14,91	33,10	18,56	25,10	5,32	41	30,65	26,59	34,71	27,76	16,76	70,84	23,90	34,13	12,87	(*) p < 0,001
	Exercises	43	47,55	43,60	51,50	45,75	25,85	94,03	40,47	53,95	12,84	43	31,34	28,31	34,38	31,29	17,18	66,39	23,58	33,73	9,86	(*) p < 0,001
	Orthopeadic insoles	43	50,81	47,66	53,95	49,99	31,39	69,99	45,75	56,51	10,23	43	30,72	27,26	34,18	27,45	14,91	70,84	23,46	37,11	11,25	(*) p < 0,001
	Mixed	39	56,87	51,07	62,67	52,30	31,21	111,64	44,59	64,76	17,89	39	27,75	24,70	30,81	25,10	15,65	46,98	19,23	38,43	9,42	(*) p < 0,001
Max sways OE (mm)	Control	41	1,33	1,25	1,42	1,32	0,96	2,36	1,11	1,51	0,27	41	1,71	1,60	1,83	1,62	1,00	2,53	1,48	1,84	0,37	(*) p < 0,001
	Exercises	43	2,54	2,35	2,73	2,39	1,57	4,05	2,26	2,84	0,63	43	1,76	1,62	1,91	1,75	1,11	3,33	1,52	2,05	0,46	(*) p < 0,001
	Orthopeadic insoles	43	2,45	2,32	2,58	2,44	1,60	3,33	2,15	2,72	0,42	43	1,60	1,50	1,70	1,57	1,07	2,53	1,37	1,82	0,33	(*) p < 0,001
	Mixed	39	2,74	2,51	2,97	2,61	1,68	4,05	2,18	3,53	0,71	39	1,56	1,42	1,71	1,62	1,06	2,45	1,11	1,88	0,43	(*) p < 0,001
Max Sways CE (mm)	Control	41	5,38	3,88	6,89	3,69	1,28	18,79	2,24	4,88	4,78	41	6,11	3,21	9,01	2,46	1,25	46,09	1,92	4,88	9,19	0,403
	Exercises	43	6,35	4,80	7,90	4,21	2,07	26,11	2,98	8,63	5,03	43	5,13	3,58	6,69	3,63	1,28	27,16	1,93	5,63	5,04	0,192
	Orthopeadic insoles	43	6,74	5,24	8,25	5,24	1,75	26,67	3,32	8,98	4,90	43	6,68	4,94	8,42	4,30	1,28	26,11	2,67	11,82	5,65	0,690
	Mixed	39	7,02	5,35	8,69	5,54	1,81	26,67	2,98	9,80	5,16	39	5,98	4,28	7,68	3,24	1,29	17,01	1,72	10,71	5,24	0,143
Min Sways OE (mm)	Control	41	0,00	0,00	0,01	0,00	0,00	0,02	0,00	0,01	0,01	41	0,01	0,00	0,01	0,01	0,00	0,02	0,00	0,01	0,01	(*) p < 0,001
	Exercises	43	0,01	0,01	0,01	0,01	0,00	0,02	0,01	0,01	0,01	43	0,01	0,00	0,01	0,00	0,00	0,02	0,00	0,01	0,01	(*) 0,003
	Orthopeadic insoles	43	0,01	0,01	0,01	0,01	0,00	0,02	0,00	0,01	0,01	43	0,01	0,00	0,01	0,01	0,00	0,01	0,00	0,01	0,01	0,101
	Mixed	39	0,01	0,01	0,01	0,01	0,00	0,02	0,01	0,01	0,01	39	0,00	0,00	0,01	0,00	0,00	0,02	0,00	0,01	0,01	(*) 0,002
Min Sways CE (mm)	Control	41	0,01	0,00	0,01	0,00	0,00	0,02	0,00	0,01	0,01	41	0,01	0,01	0,01	0,01	0,00	0,02	0,00	0,01	0,01	0,170
	Exercises	43	0,01	0,01	0,01	0,01	0,00	0,02	0,01	0,01	0,01	43	0,01	0,01	0,01	0,01	0,00	0,01	0,00	0,01	0,00	0,287
	Orthopeadic insoles	43	0,01	0,01	0,01	0,01	0,00	0,02	0,00	0,01	0,01	43	0,01	0,00	0,02	0,01	0,00	0,10	0,00	0,01	0,02	0,853
	Mixed	39	0,01	0,00	0,01	0,01	0,00	0,02	0,00	0,01	0,01	39	0,01	0,00	0,01	0,01	0,00	0,10	0,00	0,01	0,02	0,816

*OE* open eyes, *CE* closed eyes, *n* numbers, *M* mean, CI (95%) 95% confidence interval, *Me* Median, *Min* minimum, *Max* maximum, *Q1* quartile 1, *Q3* quartile 3, *SD* standard deviation, p* – p value – significant statistical values are highlighted, (*) – variables statistically significant with Bonferroni correction.

In the combined group, a significant decrease in the ellipse area of COP sway with eyes open was also observed (p < 0.05), which may indicate the most effective result of combined therapy on balance control. Furthermore, the maximum and minimum COP sway with eyes open were reduced in the exercise groups and the combined group, confirming the positive effect of stabilization training. In contrast, the control group, which received no intervention, showed deterioration in most posturographic parameters, including an increase in the length and velocity of the COP trajectory and an increase in the area of the sway ellipse.

When comparing the differences in the 6-week evaluation period between groups, the greatest reduction in statistically significant parameters was achieved in the group combining insole therapy and exercises – this is evidenced, among others, by the median values (Table 3).

**Table 3:** Assessment of changes in individual groups.

	Variable	*n*	*M*	*CI (95%)*		*Me*	*Min*	*Max*	*Q1*	*Q3*	*SD*
Control	Sway length OE (mm)	41	360,76	232,63	488,89	317,08	-308,26	1657,59	147,69	550,37	405,94
	Sway length CE (mm)	41	405,71	203,43	608,00	347,73	-677,39	2682,34	66,63	604,51	640,88
	Ellipse area OE (mm^2^)	41	146,25	91,29	201,21	94,28	-8,32	795,84	28,21	190,58	174,13
	Ellipse area CE (mm^2^)	41	49,91	0,92	98,90	56,11	-274,51	465,17	-32,04	99,75	155,22
	Mean speed OE (mm/s)	41	7,17	4,65	9,68	6,26	-6,08	32,57	3,02	10,70	7,98
	Mean speed CE (mm/s)	41	8,08	4,12	12,04	6,88	-13,32	52,09	1,57	12,45	12,55
	Max sways OE (mm)	41	0,38	0,26	0,51	0,36	-0,35	1,19	0,04	0,69	0,40
	Max sways CE (mm)	41	0,72	-2,09	3,54	-1,23	-14,27	27,30	-2,29	1,19	8,92
	Min sways OE (mm)	41	0,00	0,00	0,01	0,00	-0,01	0,02	0,00	0,01	0,01
	Min sways CE (mm)	41	0,00	0,00	0,00	0,00	-0,02	0,01	0,00	0,01	0,01
Orthopeadic insoles	Sway length OE (mm)	43	-1246,19	-1503,95	-988,42	-1246,14	-3759,61	-80,63	-1687,84	-603,31	837,58
	Sway length CE (mm)	43	-981,74	-1210,37	-753,10	-982,87	-2441,52	885,97	-1638,92	-369,40	742,92
	Ellipse area OE (mm^2^)	43	-39,28	-118,21	39,64	-30,16	-965,47	851,28	-75,62	60,57	256,45
	Ellipse area CE (mm^2^)	43	-39,38	-91,46	12,70	-2,82	-577,77	391,66	-143,10	64,31	169,22
	Mean speed OE (mm/s)	43	-23,61	-28,09	-19,13	-24,29	-57,40	-1,66	-32,78	-11,57	14,56
	Mean speed CE (mm/s)	43	-20,09	-24,49	-15,69	-21,57	-48,31	16,50	-32,33	-9,32	14,30
	Max sways OE (mm)	43	-0,85	-1,01	-0,69	-0,77	-1,94	0,11	-1,21	-0,46	0,52
	Max sways CE (mm)	43	-0,07	-2,49	2,35	-0,27	-14,85	23,03	-5,28	3,97	7,87
	Min sways OE (mm)	43	0,00	0,00	0,00	0,00	-0,01	0,01	-0,01	0,00	0,01
	Min sways CE (mm)	43	0,00	0,00	0,01	0,00	-0,02	0,10	-0,01	0,01	0,02
Exercises	Sway length OE (mm)	43	-1015,94	-1217,64	-814,24	-984,08	-2862,81	19,09	-1425,08	-512,93	655,40
	Sway length CE (mm)	43	-826,75	-1039,09	-614,42	-648,69	-2509,61	500,84	-1115,46	-415,01	689,95
	Ellipse Area OE (mm^2^)	43	24,10	-55,51	103,71	0,67	-368,57	1162,77	-72,49	78,68	258,68
	Ellipse Area CE (mm^2^)	43	3,29	-28,74	35,32	7,07	-382,31	197,38	-40,87	79,48	104,08
	Mean Speed OE (mm/s)	43	-20,04	-24,02	-16,05	-19,41	-56,40	0,46	-28,20	-10,16	12,95
	Mean Speed CE (mm/s)	43	-16,20	-20,38	-12,03	-12,79	-49,27	10,09	-21,91	-8,32	13,57
	Max Sways OE (mm)	43	-0,78	-0,98	-0,57	-0,69	-2,53	0,28	-1,18	-0,34	0,66
	Max Sways CE (mm)	43	-1,21	-3,45	1,02	-0,55	-24,82	20,09	-4,67	2,55	7,27
	Min Sways OE (mm)	43	0,00	-0,01	0,00	0,00	-0,02	0,01	-0,01	0,00	0,01
	Min Sways CE (mm)	43	0,00	0,00	0,00	0,00	-0,01	0,01	-0,01	0,00	0,01
Mixed	Sway Length OE (mm)	39	-1649,16	-2023,17	-1275,15	-1428,70	-3966,19	-67,27	-2663,66	-560,38	1153,77
	Sway Length CE (mm)	39	-1454,52	-1790,41	-1118,62	-1179,41	-4336,65	151,04	-2139,25	-698,66	1036,20
	Ellipse Area OE (mm^2^)	39	-86,68	-141,10	-32,26	-30,16	-653,90	170,56	-78,47	-9,36	167,88
	Ellipse Area CE (mm^2^)	39	-39,31	-79,18	0,56	-25,39	-392,54	214,63	-76,78	17,09	123,00
	Mean Speed OE (mm/s)	39	-32,43	-39,79	-25,07	-28,19	-78,27	-1,68	-52,13	-11,08	22,72
	Mean Speed CE (mm/s)	39	-29,12	-35,80	-22,44	-23,10	-85,07	2,91	-43,12	-13,58	20,61
	Max Sways OE (mm)	39	-1,18	-1,47	-0,89	-1,04	-2,96	0,27	-1,87	-0,45	0,89
	Max Sways CE (mm)	39	-1,04	-3,48	1,40	-1,74	-15,95	14,80	-5,86	1,16	7,51
	Min Sways OE (mm)	39	-0,01	-0,01	0,00	-0,01	-0,02	0,02	-0,01	0,00	0,01
	Min Sways CE (mm)	39	0,00	0,00	0,01	0,00	-0,02	0,10	0,00	0,00	0,02

*OE* open eyes, *CE* closed eyes, *n* numbers, *M* mean, CI (95%) 95% confidence interval, *Me* Median, *Min* minimum, *Max* maximum, *Q1* quartile 1, *Q3* quartile 3, *SD* standard deviation.

When comparing the effects between the different treatment groups, statistically significant differences were found between the control group and the other groups in the following parameters: sway length, mean center of gravity velocity with eyes closed and open, and eye surface area with eyes open. Furthermore, a statistically significant difference was found between the control group and the combined therapy group for the parameters of eye surface area with eyes closed and minimal eye sway with eyes open. In the case of the exercises and control groups, such significance was achieved in the assessment of minimal eye sway with eyes open (Table 4).

**Table 4:** P-statistic values for comparisons between groups.

		Orthopeadic insoles	Mixed	Exercises	Control
(*) p < 0,001					
Sway length OE (mm)	Orthopeadic insoles		1,000	1,000	(*) p < 0,001
	Mixed	1,000		0,439	(*) p < 0,001
	Exercises	1,000	0,439		(*) p < 0,001
	Control	(*) p < 0,001	(*) p < 0,001	(*) p < 0,001	
(*) p < 0,001					
Sway length CE (mm)	Orthopeadic insoles		0,661	1,000	(*) p < 0,001
	Mixed	0,661		0,095	(*) p < 0,001
	Exercises	1,000	0,095		(*) p < 0,001
	Control	(*) p < 0,001	(*) p < 0,001	(*) p < 0,001	
(*) p < 0,001					
Ellipse area OE (mm^2^)	Orthopeadic insoles		0,900	1,000	(*) p < 0,001
	Mixed	0,900		0,120	(*) p < 0,001
	Exercises	1,000	0,120		(*) p < 0,001
	Control	(*) p < 0,001	(*) p < 0,001	(*) p < 0,001	
p = 0,0206					
Ellipse area CE (mm^2^)	Orthopeadic insoles		1,000	1,000	0,114
	Mixed	1,000		0,526	0,025
	Exercises	1,000	0,526		1,000
	Control	0,114	0,025	1,000	
(*) p < 0,001					
Mean speed OE (mm/s)	Orthopeadic insoles		1,000	1,000	(*) p < 0,001
	Mixed	1,000		0,437	(*) p < 0,001
	Exercises	1,000	0,437		(*) p < 0,001
	Control	(*) p < 0,001	(*) p < 0,001	(*) p < 0,001	
(*) p < 0,001					
Mean speed CE (mm/s)	Orthopeadic insoles		1,000	1,000	(*) p < 0,001
	Mixed	1,000		0,075	(*) p < 0,001
	Exercises	1,000	0,075		(*) p < 0,001
	Control	(*) p < 0,001	(*) p < 0,001	(*) p < 0,001	
(*) p < 0,001					
Max Sways OE (mm)	Orthopeadic insoles		1,000	1,000	(*) p < 0,001
	Mixed	1,000		0,666	(*) p < 0,001
	Exercises	1,000	0,666		(*) p < 0,001
	Control	(*) p < 0,001	(*) p < 0,001	(*) p < 0,001	
p = 0,690					
Max sways CE (mm)	Orthopeadic insoles		1,000	1,000	1,000
	Mixed	1,000		1,000	1,000
	Exercises	1,000	1,000		1,000
	Control	1,000	1,000	1,000	
(*) p < 0,001					
Min sways OE (mm)	Orthopeadic insoles		0,460	1,000	0,105
	Mixed	0,460		1,000	(*) p < 0,001
	Exercises	1,000	1,000		(*) 0,002
	Control	0,105	(*) p < 0,001	(*) 0,002	
p = 0,255					
Min sways CE (mm)	Orthopeadic insoles		1,000	1,000	1,000
	Mixed	1,000		1,000	1,000
	Exercises	1,000	1,000		0,456
	Control	1,000	1,000	0,456	

*OE* open eyes, *CE* closed eye, p – p value—significant statistical values are highlighted, (*)—variables statistically significant with Bonferroni correction.

The KOOS questionnaire assessed quality of life, demonstrating statistically significant improvements in pain, symptoms, activities of daily living, sports and recreational activities, and quality of life across the study groups (Table 5).

**Table 5:** Functional and quality of life assessments according to the KOOS questionnaire before and after therapy in each study group.

	Group	Before										After 6 weeks										P*
		n	M	CI (95%)		Me	Min	Max	Q1	Q3	SD	n	M	CI (95%)		Me	Min	Max	Q1	Q3	SD	
Pain	Control	41	72,56	70,04	75,08	72,22	58,33	97,22	66,67	75,00	7,98	41	66,53	64,07	69,00	66,67	50,00	88,89	61,11	69,44	7,81	(*) p < 0,001
	Exercises	43	69,83	65,88	73,78	72,22	41,67	94,44	61,11	77,78	12,84	43	75,65	72,35	78,95	75,00	50,00	97,22	66,67	83,33	10,72	(*) p < 0,001
	Orthopeadic insoles	43	64,21	60,79	67,63	63,89	41,67	86,11	58,33	72,22	11,11	43	76,29	73,54	79,04	75,00	50,00	94,44	69,44	83,33	8,94	(*) p < 0,001
	Mixed	39	60,40	57,23	63,56	63,89	44,44	80,56	50,00	66,67	9,76	39	80,63	77,53	83,72	83,33	55,56	94,44	75,00	86,11	9,55	(*) p < 0,001
Symptoms	Control	41	71,78	69,31	74,24	71,43	53,57	82,14	67,86	78,57	7,82	41	64,72	62,37	67,08	64,29	50,00	78,57	60,71	67,86	7,46	(*) p < 0,001
	Exercises	43	77,08	74,28	79,88	75,00	50,00	92,86	71,43	82,14	9,10	43	83,31	80,25	86,37	85,71	50,00	92,86	78,57	92,86	9,94	(*) p < 0,001
	Orthopeadic insoles	43	74,67	71,69	77,64	78,57	53,57	92,86	67,86	82,14	9,67	43	82,56	79,90	85,22	85,71	60,71	96,43	78,57	89,29	8,65	(*) p < 0,001
	Mixed	39	71,61	68,45	74,78	71,43	53,57	89,29	64,29	78,57	9,76	39	88,92	85,95	91,89	89,29	64,29	100,00	85,71	96,43	9,15	(*) p < 0,001
ADL	Control	41	88,27	87,56	88,99	88,24	85,29	94,12	86,76	89,71	2,27	41	83,25	82,16	84,34	83,82	75,00	91,18	80,88	85,29	3,46	(*) p < 0,001
	Exercises	43	75,00	70,60	79,40	79,41	35,29	95,59	69,12	85,29	14,30	43	83,31	80,25	86,37	85,71	50,00	92,86	78,57	92,86	9,94	(*) p < 0,001
	Orthopeadic insoles	43	71,51	66,27	76,75	77,94	38,24	95,59	54,41	86,76	17,02	43	74,86	70,32	79,41	80,88	50,00	95,59	61,76	88,24	14,77	(*) p < 0,001
	Mixed	39	63,84	59,04	68,64	61,76	38,24	92,65	52,94	76,47	14,80	39	74,21	70,19	78,23	76,47	55,88	97,06	61,76	80,88	12,40	(*) p < 0,001
Sport/Rec	Control	41	45,73	43,63	47,84	45,00	30,00	60,00	45,00	50,00	6,67	41	41,34	39,14	43,55	40,00	30,00	60,00	35,00	45,00	6,98	(*) p < 0,001
	Exercises	43	45,70	42,50	48,89	45,00	25,00	65,00	35,00	55,00	10,38	43	55,12	51,77	58,47	55,00	25,00	75,00	45,00	60,00	10,88	(*) p < 0,001
	Orthopeadic insoles	43	46,16	42,56	49,76	45,00	25,00	75,00	40,00	55,00	11,69	43	54,65	51,39	57,91	55,00	30,00	85,00	45,00	60,00	10,60	(*) p < 0,001
	Mixed	39	43,21	40,29	46,12	45,00	30,00	70,00	35,00	50,00	8,99	39	65,00	61,51	68,49	65,00	40,00	85,00	60,00	70,00	10,76	(*) p < 0,001
QoL	Control	41	60,98	58,23	63,72	62,50	43,75	81,25	56,25	68,75	8,70	41	51,98	49,07	54,89	50,00	37,50	75,00	43,75	56,25	9,21	(*) p < 0,001
	Exercises	43	54,36	50,41	58,31	56,25	25,00	81,25	50,00	62,50	12,83	43	64,10	61,25	66,95	62,50	43,75	81,25	56,25	68,75	9,26	(*) p < 0,001
	Orthopeadic insoles	43	53,78	49,91	57,65	50,00	31,25	75,00	43,75	62,50	12,58	43	60,17	56,82	63,53	62,50	31,25	75,00	50,00	68,75	10,91	(*) p < 0,001
	Mixed	39	47,76	43,88	51,63	50,00	25,00	68,75	43,75	56,25	11,95	39	76,76	72,28	81,24	81,25	37,50	93,75	68,75	87,50	13,82	(*) p < 0,001

*n* numbers, *M* mean, CI (95%) – 95% confidence interval, *Me* Median, *Min* minimum, *Max* maximum, *Q1* -quartile 1, *Q3* quartile 3, *SD* standard deviation, p* – p value—significant statistical values are highlighted, (*)—variables statistically significant with Bonferroni correction.

Quality of life and functionality improved to a statistically significant level, demonstrating significant differences between groups in pain, symptom, ADL, Sport/Rec., and QoL scores across all groups. However, no significant differences were observed between the exercise and orthopedic insole groups in symptom, ADL, Sport/Rec., and QoL scores (Table 6).

**Table 6:** P-statistics for comparisons between groups in the KOOS assessment.

		Control	Exercises	Orthopeadic insoles	Mixed
(*) p < 0,001					
Pain	Control		(*) p < 0,001	(*) p < 0,001	(*) p < 0,001
	Exercises	(*) p < 0,001		0,020	(*) p < 0,001
	Orthopeadic insoles	(*) p < 0,001	0,020		0,033
	Mixed	(*) p < 0,001	(*) p < 0,001	0,033	
(*) p < 0,001					
Symptoms	Control		(*) p < 0,001	(*) p < 0,001	(*) p < 0,001
	Exercises	(*) p < 0,001		1,000	(*) p < 0,001
	Orthopeadic insoles	(*) p < 0,001	1,000		(*) 0,004
	Mixed	(*) p < 0,001	(*) p < 0,001	(*) 0,004	
(*) p < 0,001					
ADL	Control		(*) p < 0,001	(*) p < 0,001	(*) p < 0,001
	Exercises	(*) p < 0,001		1,000	(*) p < 0,001
	Orthopeadic insoles	(*) p < 0,001	1,000		(*) p < 0,001
	Mixed	(*) p < 0,001	(*) p < 0,001	(*) p < 0,001	
p = 0,0206					
Sport/Rec	Control		(*) p < 0,001	(*) p < 0,001	(*) p < 0,001
	Exercises	(*) p < 0,001		1,000	(*) 0,001
	Orthopeadic insoles	(*) p < 0,001	1,000		(*) p < 0,001
	Mixed	(*) p < 0,001	(*) 0,001	(*) p < 0,001	
(*) p < 0,001					
QoL	Control		(*) p < 0,001	(*) p < 0,001	(*) p < 0,001
	Exercises	(*) p < 0,001		1,000	(*) p < 0,001
	Orthopeadic insoles	(*) p < 0,001	1,000		(*) p < 0,001
	Mixed	(*) p < 0,001	(*) p < 0,001	(*) p < 0,001	

p – p value—significant statistical values are highlighted, (*)—variables statistically significant with Bonferroni correction.

Furthermore, in the assessment of the clinical effect of the KOOS questionnaire between the insole and exercise groups on pain assessment the significantly high effect size was obtained (Table 7).

**Table 7:** Effect size – Cohen’s d for KOOS – significant statistical values are highlighted.

Cohen’s d	Pain	Symptoms	ADL	Sport Rec	QoL
Control vs Orthopedic insoles	3,830	2,763	2,277	2,363	2,218
Control vs Exercises	2,485	2,560	2,739	2,294	2,678
Control vs Mixed	3,804	3,795	2,698	3,067	3,696
Orthopedic insoles vs Exercises	1,122	0,257	0,055	0,132	0,524
Orthopedic insoles vs Mixed	1,090	1,253	1,151	1,433	2,312
Exercises vs Mixed	1,921	1,506	1,269	1,284	1,962

Multivariate analysis using the general linear model (GLM) was performed to assess the potential influence of age and BMI as covariates. For posturographic parameters, age was not a significant covariate (Wilks’ λ = 0.923, F = 0.590, p = 0.915), whereas BMI showed a significant effect (Wilks’ λ = 0.784, F = 1.956, p = 0.013). A significant effect of group was observed in both models (p < 0.001), indicating that differences between groups were independent of age and remained significant after adjusting for BMI.

For KOOS outcomes, age was a significant covariate (Wilks’ λ = 0.854, F = 2.593, p = 0.006), while BMI was not (p = 0.102). Importantly, the group effect remained significant in both models (p < 0.001), confirming that the observed differences in patient-reported outcomes were independent of these variables.

## Discussion

The results confirm the alternative hypothesis of significant differences between the groups, with particular emphasis on greater improvement in the group receiving the combined intervention (exercises + orthopedic insoles).

Despite abundant evidence supporting the benefits of body core stability training and proper foot alignment in the kinematic chain in the treatment of KOA, there are no studies evaluating and comparing the effectiveness of both therapies—medical training programs and personalized orthopedic insoles. This study assessed the impact of an individualized rehabilitation program on balance ability, analyzed using posturography, while numerous studies have assessed balance disorders solely using clinical tests, which are subjective and dependent on the examiner’s skill. Furthermore, this study conducted a comparative analysis of the effectiveness of the therapeutic methods used. Degenerative changes associated with ageing and decreased physical fitness had a negative impact on coordination and balance, increasing the risk of falls. The introduction of appropriately selected exercises and correction of the lower limb axis contribute to reducing the pathological load on joint cartilage resulting from abnormal foot function. The essence of the conducted research was a holistic approach to the patient—not only in terms of the knee joints but also coexisting balance deficits and foot dysfunctions. Individualized exercises tailored to each patient’s needs and the use of orthopedic insoles allowed correction of foot alignment. Analyzing the impact of functional proprioceptive training and core stabilization in patients with KOA, there is a lack of sufficient scientific evidence assessing the effectiveness of this form of rehabilitation. Previous studies have rarely provided clear evidence confirming the effectiveness of proprioceptive training in KOA. Furthermore, many publications lack consistent exercise protocols and limited data on long-term treatment outcomes. There is also a shortage of randomized, controlled trials that would allow for the development of clear guidelines regarding the form and scope of proprioceptive exercises in this group of patients^39,40^.

A study conducted by Borysiuk et al. demonstrated benefits resulting from improved stability in patients. The aim of the study was to analyze the effectiveness of the Physical Recreation Program for the Elderly based on measurements of stabilization and muscle coactivation in women aged 60–70. Two equal groups were enrolled in the study: experimental and control. Both groups participated in rehabilitation exercises, with the experimental group performing them twice a week for 60 min, and the control group once a week for 60 min. The results indicated that the implementation of the six-week exercise program resulted in a reduction in variability, velocity, and indicators related to the shift of the center of pressure of the feet on the ground during exercises performed with eyes closed on an elevated surface (p = 0.001). The obtained data suggest that a properly selected rehabilitation program with appropriate frequency leads to improved functioning of the vestibular system, which is associated with proprioception, understood as an integrated process of sensory activation in the body^41^.

In this study, daily training lasting at least 60 min was used and demonstrated high effectiveness. Combined with the use of a custom-fit orthopedic insole, the training yielded greater benefits in improving stability than performing the exercises alone. Posturographic parameters such as swing length and average speed with both eyes closed and open improved, while in the mixed group, parameters assessing the area of the ellipse’s center of gravity also improved (p = 0.001). The clinical effect size showed the largest significant difference for the assessment of the length of sway with eyes open between the control and exercise groups (d = 2.512), and between the control and mixed groups for: the assessment of the length of sway with eyes closed (d = 2.172), the area of the ellipse with eyes open (d = 1.361) and the assessment of the average speed with eyes closed (d = 2.193) (Table 8), suggesting that the exercise training with the individual insert positively influenced muscle memory and the sense of proprioception compared to other methods.

**Table 8:** Effect size – Cohen’s d for postsurographic parameters – significant statistical values are highlighted.

Cohen’s d	SWAY length OE	Sway length CE	Ellipse area OE	Ellipse area CE	Mean speed OE	Mean speed CE	Max sways OE	Max sways CE	Min sways OE	Min sways CE
Control vs orthopedic insoles	2,423	1,996	0,843	0,549	2,605	2,091	2,643	0,094	0,000	0,000
Control vs exercises	2,512	1,849	0,551	0,354	2,516	1,856	2,114	0,238	0,000	0,000
Control vs mixed	2,348	2,172	1,361	0,635	2,349	2,193	2,280	0,213	1,000	0,000
Orthopedic insoles vs exercises	0,306	0,216	0,246	0,304	0,259	0,279	0,118	0,150	0,000	0,000
Orthopedic insoles vs mixed	0,403	0,529	0,217	0,000	0,467	0,514	0,458	0,126	1,000	0,000
Exercises vs mixed	0,684	0,720	0,503	0,375	0,679	0,748	0,514	0,023	1,000	0,000

*OE* open eyes, *CE* closed eyes, *Min* minimum, *Max* maximum.

Eighty-nine patients participated in the study by Ince et al. The Biodex training group, the classical training group, and the control group had global stability index values of 1.0 ± 0.07, 1.4 ± 0.07, and 1.4 ± 0.07 respectively and Modified Clinical Test of Sensory Interaction and Balance—Condition-3 values of 0.7 ± 0.04, 0.9 ± 0.04, and 0.9 ± 0.04 respectively at the end of treatment. No other significant differences between groups were found^42^.

In the present study, significant differences were obtained between the study groups and the control group without intervention in the assessment of sway length with eyes open and closed, ellipse area with eyes open, and mean velocity with eyes open and closed (p = 0.001). Moreover, statistically significant differences between groups were achieved in the assessment of the elliptical surface area with eyes closed and in the assessment of minimal oscillations with eyes open between the control and mixed groups (p = 0.001) (Table 4).

When evaluating orthopedic insoles, it should be emphasized that feet have a direct impact on balance. In Park H’s study, individuals with KOA had significantly poorer baseline balance performance in complex sensory integration tasks (p < 0.05). With textured insoles, both the healthy control and the study groups showed significant improvement, particularly in conditions requiring complex sensory integration. The OA group demonstrated a marked improvement in condition 5 (eyes closed with a reference surface for rocking) with textured insoles (p = 0.001, d = -1.043) compared to baseline. This improvement was maintained over a 3-month period without significant habituation effects. These results indicate that textured insoles are an effective intervention for improving balance control in individuals with KOA, which may be important for improving postural stability and sensory integration in this group^25^.

Similar results were also obtained in this study. Therapy with an orthopedic insole with a forefoot pronation component proved to be an effective form of rehabilitation, particularly when combined with exercise, as confirmed by the results of posturographic assessment. Therapy with an orthopedic insole alone achieved the second significant clinical effect after the combined therapy in the assessment of closed-eye sway (p = 0.001, d = 1.996), despite the relatively shorter follow-up period for both the control and study groups, which was 6 weeks.

Further studies indicate that the use of a lateral wedge insole beneficially reduces the load on the medial meniscus, which is associated with its extrusion. This load increases with increasing forces acting on the joint. After three months of therapy with lateral wedge insoles, a significant reduction in the forces acting on the medial meniscus was observed in patients with early-stage gonarthrosis, while no similar effects were observed in the control group or in the group with advanced disease. These results suggest that the lateral wedge insole is an effective solution for reducing loads on the medial meniscus in the early stages of KOA^43^.

Based on a search for the available scientific literature, direct randomized controlled trials or meta-analyses comparing orthotic inserts alone with rehabilitation exercises alone in patients with bilateral gonarthrosis, assessed by both posturography and the KOOS scale, are very limited or absent in the literature. A prospective, randomized, double-blind, controlled study by Hsieh et al. comparing rigid vs. soft lateral wedge arch support (LWAS) inserts in 90 patients with bilateral gonarthrosis used KOOS and posturography with the Biodex Stability System to assess postural stability, dynamic balance, and fall risk, as did the present study. Soft LWAS inserts have been shown to significantly improve pain (p = 0.008), daily living function (p = 0.003), sports and recreational function (p = 0.012), and quality of life (p = 0.021) significantly as compared to rigid inserts^44^.

Another RCT conducted by Dwarakanathan et al. (2022) compared an unloader knee brace vs. a lateral wedge insert in 66 patients with medial knee arthrosis (56% bilateral) using the KOOS and the Biodex Balance System, assessing stability, COP path length, mean velocity, and mobility. The knee brace improved static balance significantly more than the lateral wedge inserts (stability p = 0.0004), although both interventions resulted in significant improvements in all KOOS subscales (p < 0.05)^45^.

A systematic review with meta-analysis of 18 randomized controlled trials by Huang et al., including 944 patients, evaluated various physiotherapy and orthopedic interventions. The most effective interventions were found to be the combination of lateral wedge inserts with a knee brace for reducing the first peak knee adduction moment (KAM). Neuromuscular exercises, on the other hand, showed an increase in the first peak KAM compared to the control group^46^.

These results indicate that lack of treatment may lead to progressive deterioration of postural stability in patients with early KOA. A deterioration of stabilometric parameters was observed in the control group, whereas these parameters improved in the treatment groups. This study confirmed that the use of an orthopedic insole with a forefoot pronation element significantly improved stabilometric parameters in patients with medial knee arthrosis. The best results are achieved when this therapy is supplemented with an individually tailored training program. Combining exercises and inserts can be recommended as a standard of care for patients with early KOA.

## Conclusion

The study demonstrated that both stabilization exercises and orthotic inserts can improve postural stability in patients with early-stage KOA. The greatest improvements in posturographic parameters were observed with the combined use of both methods, suggesting a potential synergistic effect. These results also suggest that these interventions can be safely used as part of conservative treatment.

Improvements in postural control (COP) parameters were observed, but the impact of these interventions on patients’ functional mobility and fall risk requires further study, particularly with longer-term follow-up. Due to their relatively low cost, availability, and ease of implementation, these approaches may be a valuable adjunct to therapeutic management in patients with early KOA, although their widespread use requires further confirmation in studies involving larger patient groups.

### Limitation, recommendations and generalization

The limitations of the study include the possible influence of individual factors, such as the current psychophysical condition of the participants, level of fatigue, mood or degree of concentration on the day of the study, which could have influenced the results of functional tests and posturographic parameters. These factors were not controlled or assessed using standardized tools, so future studies should consider using additional questionnaires regarding participants’ well-being, level of wakefulness, and mental and physical fatigue.

Another limitation was the lack of a detailed assessment of the participants’ daily physical activity levels. Differences in activity levels can affect both balance ability and adaptation to therapeutic interventions. Taking this factor into account in future analyses could allow for a more accurate interpretation of the obtained results.

The short follow-up period and the lack of a long-term assessment of the durability of the treatment effects were also significant limitations. The obtained results refer only to short-term effects; therefore, it is not possible to clearly determine how long the effect of stabilization exercises and orthopedic insoles on postural control lasts. It should be emphasized, however, that the study was preliminary in nature, and continued patient follow-up was planned three and six months after the intervention. This will allow for the assessment of the durability of the therapeutic effects and their potential long-term clinical significance.

Other limitations may include the relatively small sample size and the lack of consideration of other potential factors influencing balance, such as comorbidities, medications, or previous rehabilitation experience. These factors may be a source of variability in results and should be more broadly considered in future research projects.

At the same time, a strength of this study was the use of both posturographic and clinical assessment, which can find practical applications not only in specialist settings but also in the daily practice of primary care physicians and physiotherapists. This approach may support earlier detection of balance disorders in patients in the early stages of gonarthrosis and enable faster implementation of therapeutic measures.

## Data Availability

The datasets generated and analyzed during the current study and additional materials describing the intervention and comparator are available from the corresponding author on reasonable request.

## Abbreviations

KOA: Knee osteoarthritis
KOOS: Knee injury and osteoarthritis outcome score
ADL: Activities of daily living
QoL: Quality of life
ROM: Range of motion
BMI: Body mass index
COP: Center of pressure
RCT: Randomized, controlled trial
OA: Osteoarthritis
ACR: American college of rheumatology
SD: Standard deviation
IQR: Interquartile range
GDPR: General data protection regulation
n: Numbers
CI (95%): 95% Confidence interval
Q1: Quartile 1
Q3: Quartile 3
OE: Open eyes
CE: Closed eyes
LWAS: Lateral wedge arch support
KAM: Knee adduction moment
